# Effects of long-term exposure of gelatinated and non-gelatinated cadmium telluride quantum dots on differentiated PC12 cells

**DOI:** 10.1186/1477-3155-10-4

**Published:** 2012-01-20

**Authors:** Babu R Prasad, Gillian Mullins, Natalia Nikolskaya, David Connolly, Terry J Smith, Valérie A Gérard, Stephen J Byrne, Gemma-Louise Davies, Yurii K Gun'ko, Yury Rochev

**Affiliations:** 1 National Centre for Biomedical Engineering Science, National University of Ireland Galway, Galway, Ireland; 2 CRANN and the School of Chemistry, Trinity College Dublin, Dublin 2, Ireland

**Keywords:** CdTe Quantum Dots, Differentiated PC12 cells, Cytotoxicity, Neuronal Growth Factor, Apoptosis

## Abstract

**Background:**

The inherent toxicity of unmodified Quantum Dots (QDs) is a major hindrance to their use in biological applications. To make them more potent as neuroprosthetic and neurotherapeutic agents, thioglycolic acid (TGA) capped CdTe QDs, were coated with a gelatine layer and investigated in this study with differentiated pheochromocytoma 12 (PC12) cells. The QD - cell interactions were investigated after incubation periods of up to 17 days by MTT and APOTOX-Glo Triplex assays along with using confocal microscopy.

**Results:**

Long term exposure (up to 17 days) to gelatinated TGA-capped CdTe QDs of PC12 cells in the course of differentiation and after neurites were grown resulted in dramatically reduced cytotoxicity compared to non-gelatinated TGA-capped CdTe QDs.

**Conclusion:**

The toxicity mechanism of QDs was identified as caspase-mediated apoptosis as a result of cadmium leaking from the core of QDs. It was therefore concluded that the gelatine capping on the surface of QDs acts as a barrier towards the leaking of toxic ions from the core QDs in the long term (up to 17 days).

## Background

Quantum Dots (QDs) represent an attractive diagnostic and therapeutic tool, however they possess the major disadvantage of being inherently cytotoxic, due to their cadmium components [1,2]. Cellular interaction with QDs is dependent on a variety of physicochemical parameters, including size, chemical composition, surface structure, solubility, shape and aggregation; all of which can influence or modify cellular uptake [3]. There is an inverse relationship between the size of QDs and their number of surface atoms or molecules that determines the material reactivity, which is the key to defining the chemical and biological properties of QDs [3,4]. The small size of QDs also gives them the ability to traverse cell membranes and possibly the blood-brain barrier, which cannot be achieved using conventional dyes, making their use as therapeutic tools an intriguing possibility. The size of QDs is fundamental to their cellular interaction and has to be considered while studying their toxicity and distribution in various cell compartments [5]. When coated with certain biocompatible polymers, QDs have been shown to be far less toxic to cells and living organisms in the short term [6]. A fundamental problem of QDs is that of aggregation and accumulation, which are particularly prevalent upon entrapment in organelles such as vesicles, endosomes and lysosomes inside living cells [7-9]. However, little information is known about the interactions of QDs with intracellular proteins and transportation methods of QDs inside living cells [10]. Even cell-penetrating peptides such as poly-arginine and TAT, when conjugated with QDs, still become trapped within vesicles and endosomes, therefore inhibiting their use as molecular diagnostic and therapeutic targeting tools [11,12]. Notably, accumulation of QDs over longer exposure periods of 8-24 hours results in a degradation of their coatings, leading to a leakage of their toxic core particles or ions [8,13]. This core leakage has been shown to initiate the formation of reactive oxygen species (ROS), which are the key mediators in cell organelle damage and destruction. The high surface area to volume ratio of the QDs also lends itself to enhanced numbers of ROS sites [3]. Overload of Cd^2+ ^and ROS in the mitochondria leads to permeability of the inner mitochondrial membrane. Cytochrome c is then released from mitochondrial intermembrane space which then activates the downstream caspases 9 and 3, finally causing cell death by apoptosis [2,14-17].

There has been significant advancement and progress in biological imaging, especially using fluorescent semi-conductor nano-crystals due to their resistance to photo-bleaching [18-20]. This has paved the way for the development of medical diagnostics and drug delivery tools utilising QDs. One of the most important criteria for the future development of QDs as efficient cellular delivery, labelling and targeting agents is that their intracellular uptake depends on the selective detection of one molecule, or a small number of molecules. The QD probes must be able to selectively access various sub-cellular compartments which need to be targeted in order to understand the dynamics of cellular organisation without causing a cytotoxic effect during the time period required [21].

Currently, methods to access single molecule properties in living cells are limited due to the size of the probe or photo-bleaching of fluorescent biomarkers. QDs have great potential as fluorescent probes thanks to their sizes, which can range from approximately 2 to 5 nm and their enhanced photo-stability, whereby signal detection is not diminished even after exposure to the acidic cell environment [22].

Previously, we have investigated the cytotoxicity of QDs by analyzing the outcome of co-incubating a range of concentrations of various types of QDs with non-differentiated PC12 cells [23]. In this paper, we have studied the long-term cytotoxicity and localisation of gelatinated (gel) and non-gelatinated (non-gel) QDs of various sizes in differentiated PC12 cells. When treated with nerve growth factor (NGF), PC12 cells become differentiated and have functional properties enabling them to behave in a manner similar to neuronal cells [24]. Their phenotype may not be similar to primary nerve cells as their origin is from tumour cells, however, in the presence of NGF, they have the ability to produce neurites, synthesize neurotransmitters and receptors and exhibit the electrical activity, which are characteristic of neurons [25]. Although some cytotoxicity studies of QDs have been carried out with PC12 cells [26,27], in this study we clearly analyze the viability, cytotoxicity and apoptosis at different time periods and discuss the effect of exposure of QDs on PC12 cells before and after the neurites were grown. The apoptotic process involved in the cell death, as well as the intrinsic behaviour of QDs upon uptake by the cells is also analyzed.

## Results

Our aim was to analyze the effect of CdTe QDs on cell behaviour and morphology and to investigate any alterations of cell proliferation, cytotoxicity, viability and apoptosis using pre-determined assays. PC12 cells were exposed to QDs over extended co-incubation periods before and after the formation of neurites. Stock solutions of gel and non-gel QDs (10^-4 ^M) [28] were diluted to 10^-9 ^M and incubated with the cells as described in the experimental section.

### 1. **Characterisation of CdTe QDs**

All types of QDs used in this study were fully characterised prior to their biological testing. UV-Visible absorption spectroscopy and photoluminescence emission spectroscopy provided information on their exciton band, core diameter, emission wavelength and quantum efficiency. These properties are summarized in Table 1 for all four types of QDs. Due to the presence of carboxylic groups on the surface of the particles, they were negatively charged and stable in basic pH solutions.

**Table 1:** Characteristics of QDs

QD type	Surface	Absorbance peak (nm)	PL emission peak (nm)	Quantum Yield	Size(nm) (+/- 0.1)	Hydrodynamic diameter (nm)	Zeta potential (mV)
**Red non-gel**	TGA	586	608	30%	4.7	11.7	-30
**Orange non-gel**	TGA	515	546	23%	2.4	3.6	-27
**Red gel**	TGA-gelatine	579	610	34%	4.5	14.3	-29
**Orange gel**	TGA-gelatine	522	550	29%	2.6	5.3	-42

### 2. **Uptake of QDs and their effect on cell morphology of differentiated PC12 cells**

**Figure 1:**
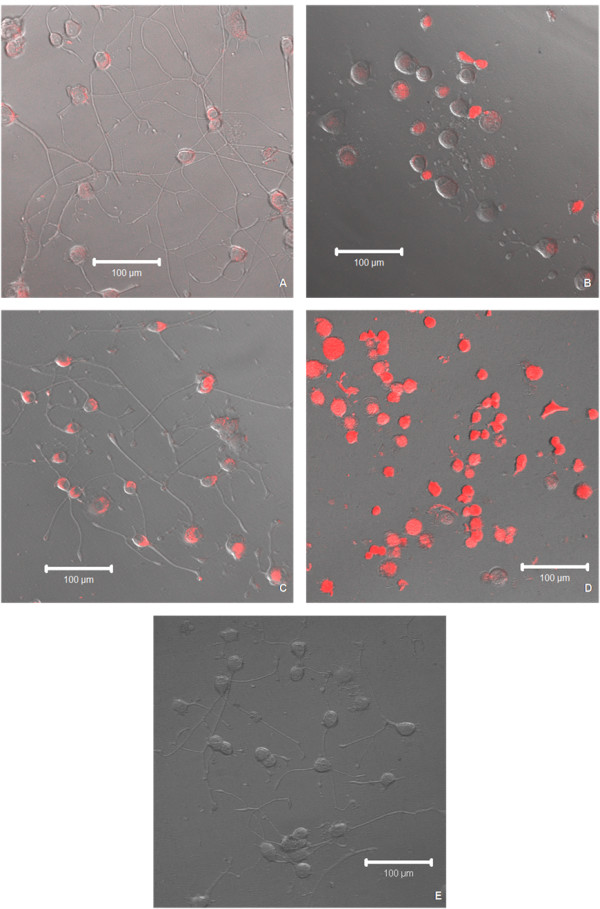
**Live confocal images**. Differential interference contrast (DIC) images of differentiated PC12 cells with overlaid corresponding fluorescent confocal images exposed to 10^-9 ^M concentrations of QDs showing red gel QDs in (A), red non-gel QDs in (B), orange gel QDs in (C), orange non-gel QDs in (D) and the control in (E) without exposure to QDs following 14 days of co-incubation [scale bar = 100 μm].

**Figure 2:**
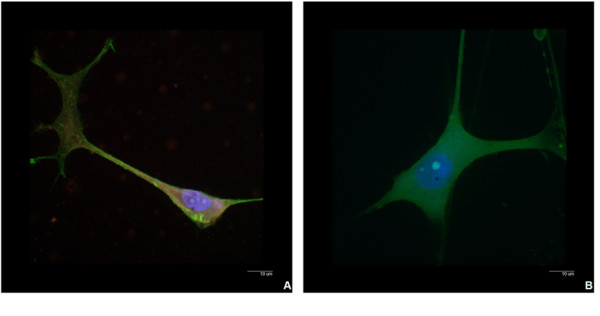
**Stained confocal images**. Overlaid fluorescent confocal images illustrating the morphology of the actin stained differentiated PC12 cells exposed to the red gel QDs (A) and differentiated PC12 cells without exposure to QDs as a control (B) following 17 days of co-incubation [Scale bar = 10 μm].

**Figure 3:**
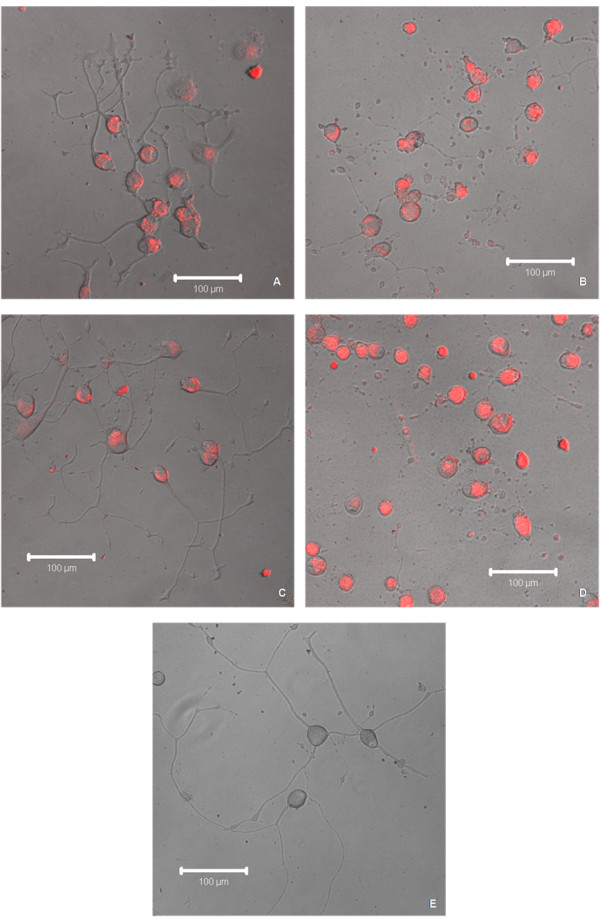
**Live confocal images**. Differential interference contrast (DIC) images of differentiated PC12 cells with overlaid corresponding fluorescent confocal images treated with NGF for 6 days prior to exposure to 10^-9 ^M concentrations of QDs showing red gel QDs in (A), red non-gel QDs in (B), orange gel QDs in (C), orange non-gel QDs in (D) and control in (E) without exposure to QDs following 7 days of co-incubation [scale bar = 100 μm].

Confocal images were taken to visually inspect QD uptake, localisation and cell morphology following exposure to QDs before and after the differentiation of PC12 cells (Figures 1, 2 and 3).

As seen in Figure 1, the QDs were found to be located within the cytoplasm of differentiated PC12 cells in all the images. The cells exposed to gel QDs (red and orange) (panels A and C respectively) exhibited a similar morphology and neurite growth to the control (no treatment of QDs) in panel E. The cells exposed to non-gel QDs (panels B and D respectively) appeared rounded with partial inhibition of neurite growth (red non-gel) or no neurite growth (orange non-gel). Cell morphology changes are attributed to the absence of the protective gelatinated shell. These cellular morphologies indicated that the presence of gelatine provides a protective surface coating for the QDs and prevents the initiation of deleterious effects on the morphology and cellular activity of differentiated PC12 cells.

In Figure 2, the nucleus was stained with DAPI (blue) and the cytoplasm was actin stained (green). The gel QDs (red luminescence) in panel A are visible predominantly in the cytoplasm and their presence, even after 17 days of co-incubation, did not seem to significantly perturb the cells. The QDs were also parsimoniously distributed in the neurites. The cell morphology did not change compared to the controls in panel B.

Figure 3 shows the overlaid differential interference contrast (DIC) images with corresponding fluorescent images of the differentiated PC12 cells treated with NGF for 6 days prior to exposure to 10^-9 ^M concentrations of the QDs. Red gel QDs are shown in panel A, red non-gel QDs in panel B, orange gel QDs in panel C and orange non-gel QDs in panel D following 7 days of co-incubation. The QDs were found to be located within the cytoplasm of differentiated PC12 cells in all the images. The cells exposed to red and orange gel QDs (panels A and C respectively) showed similar morphology and neurite growth compared to the control (no treatment of QDs) in panel E. There was evidence of slight neurite degeneration in the cells exposed to orange gel QDs more so than in the cells exposed to red gel QDs, illustrating that the red gel QDs are more cyto-protective than the orange gel QDs. The cells exposed to red non-gel and orange non-gel QDs (panels B and D respectively) appeared rounded with partial degeneration and full degeneration (fragmentation) of neurites respectively, which suggests that orange non-gel QDs are more cytotoxic than red non-gel QDs which is expected due to the enhanced cytotoxicity of the smaller orange QDs relative to their larger red counterparts.

These initial observations using confocal microscopy illustrate the effect of exposure of QDs before and after the differentiation of PC12 cells on cell survival and morphology. In order to further investigate the cell behaviour, several assays were used to study the effect on cell proliferation, cytotoxicity, viability and apoptosis.

### 3. **Effect of QDs on cellular activity of differentiated PC12 cells**

Results were analysed using a one-way ANOVA analysis by Tukey's mean comparison, where results with a p-value of less than 0.05 were reported as statistically significant and their occurrence can be deemed to be due to interactions in the system under investigation and chance variation can be eliminated. MTT (cell proliferation) and APOTOX Triplex (cytotoxicity, viability and apoptosis) assays were run to analyze the effect of different QD types and size following exposure of QDs before and after the differentiation of PC12 cells.

#### MTT Assay

**Figure 4:**
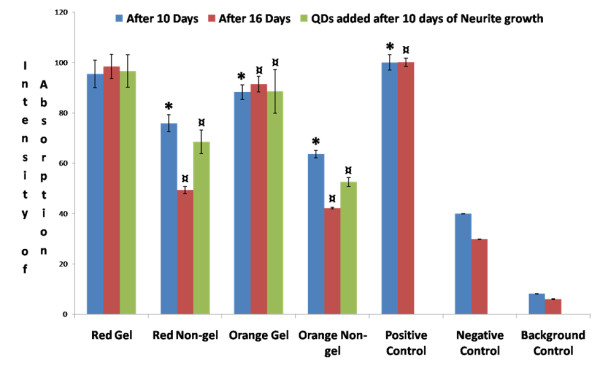
**Proliferation assay**. Graph of MTT assay after 10 and 16 days showing the rate of proliferation of differentiated PC12 cells after exposure to concentrations [10^-9 ^M] of the gel and non-gel QDs. Positive control shows differentiated PC12 cells without exposure to QDs; the graph also shows differentiated PC12 cells which were exposed with red and orange QDs of gel and non-gel types after neurites were grown for 10 days. Symbols * and ¤ denote examples of statistical significance in comparison with positive controls using a one-way ANOVA (P < 0.05) by Tukey's mean comparison.

The graph in Figure 4 depicts the results of an MTT assay for PC12 cells treated with NGF and exposed to QDs after periods of 10 and 16 days. After 10 and 16 days, the proliferation of cells exposed to red gel QDs was the same as the positive controls whereas the proliferation of cells exposed to smaller (orange) gel QDs was significantly reduced. This clearly showed that smaller orange gel QDs are significantly more toxic than larger red gel QDs towards differentiated PC12 cells. Similarly, smaller orange non-gel QDs appeared to be significantly more cytotoxic than larger red non-gel QDs as co-incubation periods were prolonged. Overall, gel QDs were found to be less cytotoxic than their non-gel counterparts. The absorption of MTT, and therefore cell proliferation, further decreased when the co-incubation time was extended up to 16 days. Even after prolonged exposure time, smaller QDs had a higher adverse effect on cell proliferation compared to their larger counterparts. As observed from the results displayed above, the gelatine layer on the surface of the gel QDs regardless of size proved to effectively reduce their cytotoxicity. This suggests that cell toxicity of QDs is due to the leakage of cadmium ions or from reactive oxygen species as we discussed in our previous paper with non-differentiated PC12 cells [23].

#### ApoTox-Glo™Triplex Assay

**Figure 5:**
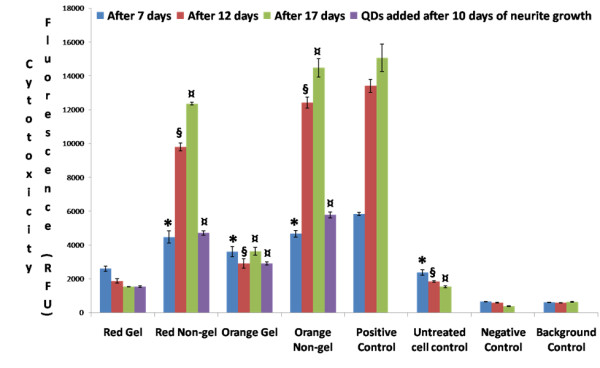
**Cytotoxicity assay**. Graph of APOTOX GLO Triplex assay showing the cytotoxicity of differentiated PC12 cells after 7, 12 and 17 days treated with red and orange QDs of gel and non-gel types along with controls. The cells were also treated with red and orange QDs of gel and non-gel types and neurites were subsequently grown for 10 days. Symbols *, § and ¤ denote examples of statistical significance in comparison with untreated cell controls using a one-way ANOVA (P < 0.05) by Tukey's mean comparison.

The graph in Figure 5 depicts the results of an APOTOX- Glo Triplex assay showing the cytotoxicity of PC12 cells treated with NGF and exposed to red and orange QDs of gel and non-gel types along with controls. The cells were also similarly treated after the neurites were grown for 10 days. After periods of 7, 12 and 17 days, the cytotoxicity of red gel QDs was comparable to the untreated cell controls but in the case of smaller gel QDs the cytotoxicity increased significantly. This clearly showed that smaller orange gel QDs are significantly more toxic than the larger red gel QDs. The smaller orange non-gel QDs also exhibited significantly higher cytotoxicity than the larger red non-gel QDs as co-incubation periods were prolonged, and non-gel QDs were more cytotoxic than gel ones. Cytotoxicity levels increased as the co-incubation periods were prolonged up to 17 days. Gel and non-gel QDs exhibited the same trend with regards to the impact of particle size on cytotoxicity, with the smaller ones being the more toxic. Furthermore, the cells exposed to non-gel QDs were found to be more affected than those exposed to gel QDs. We found the same trend of cytotoxicity after neurites were grown for 10 days prior to QD exposure. This shows that there is absolutely no inhibition of cellular interactions with QDs after the cells were grown with neurites.

**Figure 6:**
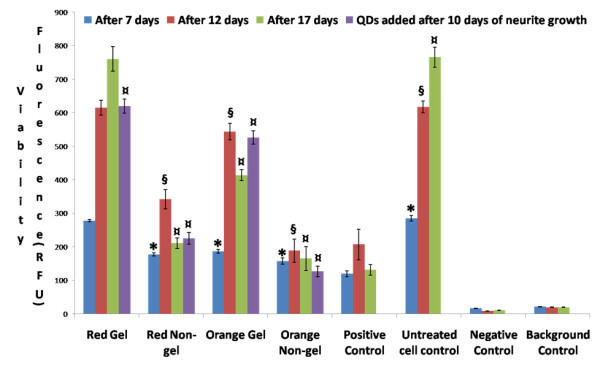
**Viability assay**. Graph of APOTOX GLO Triplex assay showing the viability of differentiated PC12 cells after 7, 12 and 17 days treated with red and orange QDs of gel and non-gel types along with controls. The cells were also treated with red and orange QDs of gel and non-gel types and neurites were subsequently grown for 10 days. Symbols *, § and ¤ denote examples of statistical significance in comparison with untreated cell controls using a one-way ANOVA (P < 0.05) by Tukey's mean comparison.

The graph in Figure 6 depicts the results of an APOTOX Triplex assay showing the viability of PC12 cells treated with NGF and exposed to red and orange QDs of gel and non-gel types along with controls and the viability of cells treated with red and orange QDs of gel and non-gel types after neurites had been grown for 10 days. After periods of 7, 12 and 17 days, the viability of cells exposed to red gel QDs was the same as that of untreated control cells, however the viability of cells exposed to the smaller orange gel QDs decreased significantly. Similar to previous assays, cells exposed to the smaller orange QDs were significantly less viable than cells exposed to the larger red QDs (both gel and non-gel) as co-incubation periods were prolonged. Viability levels decreased as the co-incubation periods were prolonged up to 17 days, and retained the same trend with regards to gel/non-gel and size influence. The cells exposed to gel QDs were found to be more viable than the ones exposed to non-gel QDs and were equally viable as untreated controls (negative controls). We found the same trend of cellular viability after neurites were grown for 10 days and cells were subsequently treated with QDs. This also shows that there is absolutely no inhibition of cellular interactions with gel QDs after the cells were grown with neurites.

**Figure 7:**
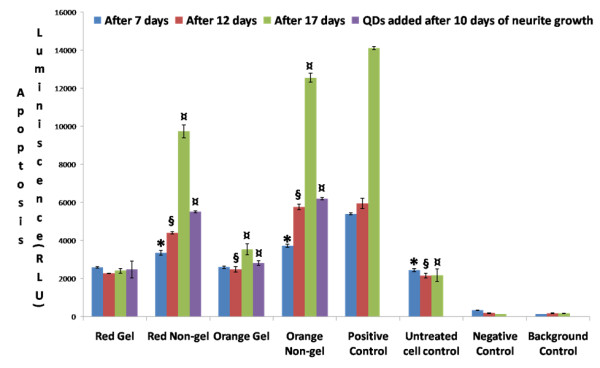
**Apoptosis assay**. Graph of APOTOX GLO Triplex assay showing the apoptosis of differentiated PC12 cells after 7, 12 and 17 days treated with red and orange QDs of gel and non-gel types along with controls. The cells were also treated with red and orange QDs of gel and non-gel types and neurites were subsequently grown for 10 days. Symbols *, § and ¤ denote examples of statistical significance in comparison with untreated cell controls using a one-way ANOVA (P < 0.05) by Tukey's mean comparison.

The graph in Figure 7 depicts the results of an APOTOX Triplex assay showing the apoptosis of PC12 cells treated with NGF and exposed to red and orange QDs of gel and non-gel types along with controls and also the apoptosis of cells which were treated with red and orange gel and non-gel QD types after neurites had been grown for 10 days. After periods of 7, 12 and 17 days, the apoptotic activity of cells exposed to red gel QDs was the same as that of untreated control cells, whereas the apoptotic activity of cells exposed to smaller orange gel QDs had significantly increased. This illustrated that smaller orange QDs were significantly more cytotoxic than the larger red QDs for both gel and non-gel QDs as co-incubation periods were prolonged. Overall, non-gel QDs induced more apoptosis than gel QDs. Apoptotic activity levels increased with both gel and non-gel QDs as the co-incubation periods were prolonged up to 17 days and retained the same trend with regards to gel/non-gel and size influence. The cells exposed to non-gel QDs were found to undergo more cell death than the cells exposed to gel QDs. We found the same trend of cell death after neurites were grown for 10 days and subsequent treatment of the cells with QDs. This shows that there is absolutely no inhibition of cellular interactions with gel QDs after the cells were grown with neurites.

## Discussion

The present study is aimed at defining the effect of gelatinated CdTe QDs on differentiated PC12 cells. The cellular uptake of QDs is mediated by proteins such as clathrins, which are coated to membrane vesicles on the cell surface at the entry [29-31]. Non-specific binding occurred less frequently for PC12 cells [32] when compared to other cells like neuroblastoma cells as studied by Gomez *et al*. [33].

Confocal microscopy has been used to identify the localisation of the particles after cellular uptake, as shown in Figure 1 and 2. Similarly to previously reported non-differentiated PC12 cells [23], gel QDs were mostly found in the cytoplasm, which became largely illuminated. This may be easily explained by the nature of the nanoparticles. The TGA-capped CdTe QDs used in this study were negatively charged thanks to the de-protonated carboxylic groups of the TGA molecules and they exhibit an average zeta potential of -40 mV. It has been shown in previous studies that negatively charged QDs have a strong tropism to core histones and histone-rich cell organelles [10]. This research suggested that the surface charge of these nanoparticles may ultimately determine their cellular uptake and therefore their location within the cell. It has been suggested that the negatively charged QDs are drawn towards the nucleus due to molecular interactions with positively charged histones. This may explain why the majority of TGA-capped CdTe QDs reside in the cytoplasm [5], surrounding the nucleus as opposed to the neurites.

Macromolecules, such as proteins and RNA, responsible for genome structure and function must be transported by selective, energy-dependent mechanisms from the cytoplasm to the nucleus. The karyopherin family of proteins maintain this process of selective import and export into the nucleus and cytoplasm. The nuclear localisation signals, nuclear transport receptors and the proteins in the nuclear pore complex ensure that no unwanted molecules are transported into the nucleus [34]. This selective transport system could be the reason why QDs are not localised within the nucleus. A second reason why QDs seem to localise only in the cytoplasm could be due to entrapment within cell organelles such as endosomes, lysosomes and vesicles. However, examination of images of differentiated PC12 cells (Figures 1 and 2), shows some localisation of QDs within the neurites. This would mean that not all the QDs are accumulated within these cell organelles, but still are not observed within the nucleus [12,31].

Figure 3 displays a comparison of the morphological changes induced by exposure of cells to QDs of different sizes and structure. The degeneration of neurites observable mostly in the case of non-gel QDs was attributed to neuronal cell death and direct axonal toxicity, as evidenced by the study of Sanjeev Kumar Mahto *et al*., with differentiated PC12 cells inside microfluidic devices [26]. Another study also showed that the degeneration of neurites was due to autophagosomes or lysosomes produced in the cell cytoplasm and in the neurites, which traverse in both anterograde and retrograde directions to destroy the already impaired mitochondria due to the toxicity of QDs [35].

Although observation of the cell morphology gave a rather clear idea of the trend in cytotoxicity among the different types of QDs, quantitative assays of the metabolic activity could provide a better understanding of the mechanisms involved. The MTT proliferation assay was designed to probe the activity of reductase enzymes as a measure of cell viability and proliferation. The results shown in Figure 4 indicated that gel QDs (both red and orange) did not significantly affect cell proliferation as compared to untreated control cell cultures. Non-gel QDs, however, caused a reduction of about 50% in cell proliferation. Interestingly, whether the cell differentiation occurred simultaneously or prior to QD treatment did not change the outcome of the assay. The MTT assay correlated well with the viability part of the APOTOX GLO Triplex assay (Figure 6), although the latter gave more subtle results, showing a discrepancy between red and orange QDs. This assay essentially assessed the cell membrane integrity and is therefore more sensitive than MTT which measures the enzyme level. Orange QDs are smaller in size and appeared to be slightly more cytotoxic then their larger red counterparts. It was previously reported by Lovric *et al*. [5] that QD cytotoxicity was inversely related to their size due to the fact that smaller particles may enter cells more readily thus interfering to a higher degree with the cell machinery. As expected, the cytotoxicity part of the APOTOX GLO Triplex assay produced similar results (Figure 5); gel QDs appeared to be much less cytotoxic than non-gel QDs, and orange (smaller) ones were more cytotoxic than red (larger) ones.

A recent study on the toxicity of QDs with PC12 cells has shown involvement of reactive oxygen species (ROS) [5] and the most common pathways involved in relation to toxicity of QDs with ROS has been discussed previously [23]. In the cell, mitochondria are cellular factories for the production of Adenosine Triphosphate (ATP) and are also a prime source of ROS production. In addition, they help to regulate the cytoplasmic calcium levels, pH and apoptosis. Abnormally increased levels of ROS (oxidative stress) during ischemia make it difficult for the neuronal cells to survive due to overwhelming multiple buffering mechanisms of ROS [15]. Oxidative stress is a state in which glutathione (GSH) is depleted with accumulation of oxidized glutathione (GSSG) [3,36]. Lower levels of ROS are easily neutralized by generation of GSH and antioxidant enzymes. Protective or injury responses in the cells are characterized by the drop in GSH/GSSG ratio [3,36-39]. At lower oxidative stress, cellular redox hemostasis occurs, intermediate oxidative stress leads to inflammation and higher oxidative stress leads to cytotoxicity which finally leads to apoptosis [3,36].

Apoptosis is one form of cell death which involves the cell death machinery, Caspase-9, Apaf-1 and Cytochrome c. Chromatin margination along the nuclear membrane, nuclear condensation, budding and fragmentation are some of the features of apoptosis which can be seen in the cell morphology. DNA fragmentation, which is one of the hallmarks of apoptosis is thought to be induced by cadmium. Cadmium toxicity is thought to affect the cells by the production of ROS and can induce apoptosis through a mitochondrial caspase dependent pathway [40]. Caspases, a family of cysteine proteases, carry out these complex biochemical events which cause cell morphology changes. Caspases are made up of initiator caspases such as caspases-8, -9 and -12, whose function is to activate downstream caspases, and executor caspases, such as caspases-3, -6 and -7, their function being to degrade cellular protein [1,41].

In previous research on human neuroblastoma cells, Chan *et al*. described the apoptotic chain of events induced by CdSe QDs through the mitochondrial release of cytochrome c and activation of caspase-9 and caspase-3 [14]. The trigger is the intracellular degradation of QDs, which leads to the release of free cadmium ions (Cd^+2^) inside the cytoplasm. These free cadmium ions inside the cells are responsible for the formation of ROS, leading to oxidation of the phospholipid Cardiolipin, which helps in associating the cytochrome c with inner mitochondrial membrane [42]. Due to oxidation of cardiolipin, cytochrome c is released, an important event in apoptotic signaling [43]. Release of cytochrome c is also due to ROS-induced changes in the conformation of the adenine nucleotide translocase, a protein which is involved in the formation of the mitochondrial permeability transition pore [44], and the voltage-dependent anion channel-selective permeabilization of the mitochondrial outer membrane [45]. It is thought that this release of Cytochrome *c *into the cytosol leads to Caspase-9 activation by Cytochrome c/Apaf-1 complex. Caspase-9 is the upstream caspase in the mitochondria-dependent apoptosis pathway and activates Caspase-3. In our study, the apoptosis assay, while confirming the general trend among the various types of QDs, provided valuable information about the mechanisms involved in cell death upon QD treatment (Figure 7). The assay itself is based on the measurement of the activity of caspase 3/7 as an indicator of apoptosis. Therefore it can be concluded that QDs, in particular non-gel types, cause cell death *via *cadmium-induced mitochondrial release of cytochrome c and activation of caspase-3 leading to apoptosis [41].

Long term exposure (up to 17 days) of PC12 cells to QDs both before and after undergoing differentiation displayed dramatic differences between non-gel and gel QDs. While the former exhibited a dramatic increase in cytototoxicity as measured by MTT and APOTOX GLO Triplex assays (Figures 4 to 7), the latter remained at a comparable level of toxicity as after 7 and 12 days of incubation. It was therefore concluded that the gelatine coating durably stabilized the QDs and created virtually no interference with cell functions over significant periods of time.

The results presented here are consistent with our previously published findings on non-differentiated PC12 cells [23]. Differentiated PC12 cells mimic neuronal cells behaviour, thus providing a model for QD interaction with neurons. The accumulation of nanoparticles in neurites was minor compared to the rest of the cytoplasm and did not appear to disturb the cell functions any further, even over extended periods of time (up to 17 days). In addition, we found that QDs did not affect differentiation itself, as proved by the growth of neurites in their presence.

## Conclusion

There is clear evidence from MTT and APOTOX-Glo Triplex assay (Cytotoxicity, Viability and Apoptosis) and also from microscopic images that the gelatine-coating helps to reduce the toxicity of CdTe QDs and assists in protecting the cells themselves. This was observed indiscriminately when neurites were grown prior to or after exposure to QDs. The difference in toxicity and resulting cell death between the orange and red gel QDs is due to the smaller size of the orange QDs. By preventing leakage of cadmium ions from the QD core and providing a biocompatible interface, the gelatine coating helps to delay caspase activation events that eventually lead to apoptosis. Gel QDs were shown neither to inhibit cell differentiation nor to be any more cytotoxic towards neuron-like differentiated cells than non-differentiated ones. This provided a good indication that these particles can remain in healthy and sensitive tissue for several days (up to 17 days) without damaging it, which opens applications in diagnostics and targeted drug delivery. This is an important starting point that can be used for development of other non-toxic nanoparticle-gelatine composites, which might have a range of potential biomedical applications such as controlled drug delivery, *in vivo *and *in vitro *diagnostics and anticancer therapy.

## Materials and methods

### Chemicals and Reagents

PC12 cells (cell line derived from a pheochromocytoma of the rat adrenal medulla) were used for this study. Dulbecco's Modification of Eagle Medium (DMEM) (Sigma-Aldrich) supplemented with 10% heat inactivated horse serum, 5% fetal bovine serum, 1% penicillin-streptomycin and Trypsin-EDTA solution and all chemicals for QD synthesis were purchased from Sigma-Aldrich. Al_2_Te_3 _was purchased from Cerac Inc. Mouse Nerve Growth Factor (mNGF 2.5S Grade 2) was purchased from Alomone labs. MTT assay to measure cell proliferation, MTT Reagent and stop solution was kindly received from Dr. Afshin Samali Group of NCBES, NUI Galway. APOTOX-Glo™ Triplex assay kit to measure cytotoxicity, viability and apoptosis was purchased from Promega Corporation. Permonax four-well chamber slide (Lab-Tek, Nalgene Nunc International), Phalloidin-FITC (Sigma-Aldrich), DAPI (Vector Laboratories), 96-well flat tissue culture plates were purchased from Sarstedt.

### Quantum Dot Synthesis

Note: all values denoted are initial concentrations and synthesis follows previously published procedures [46,47]. Millipore water (150 ml) was degassed by bubbling argon through it for approximately 1 hour. Cd(ClO_4_)_2_•6H_2_O and 1.3 molar equivalents of thio-glycolic acid (TGA) stabilizer were added to the water and the pH was adjusted to 11.2-11.3 by the addition of a 2 M NaOH solution. For samples containing gelatine, 0.3 g was added to the reaction mixture. H_2_Te gas was generated from Al_2_Te_3 _(0.25 molar equivalents as compared to cadmium per-chlorate) *via *drop-wise addition of a 0.5 M H_2_SO_4 _solution and was bubbled through the cadmium/thiol solution under a slow argon flow for approximately 10 minutes. Note: 100% reaction and carryover is assumed and cadmium is always in excess for this experiment. The resultant, non-luminescent solution was then heated to reflux (at 130°C). Following the reflux process, fractions were precipitated *via *the addition of isopropanol and were stored at 4°C. The stock solutions were further purified on a Sephadex G25 column. A Shimadzu UV-1601 UV - Visible Spectrophotometer was used to measure QD absorption while a Varian - Cary Eclipse Fluorescence Spectrophotometer was used to determine the fluorescence emission/photoluminescence (PL) spectra of QDs. Throughout the text, gel and non-gel refer to the presence of gelatine during the synthesis of the QDs. Smaller QDs (~2.5 nm) are referred to as orange QDs and larger ones (~ 4.5 nm) as red QDs. Hydrodynamic diameters and zeta potentials were measured on a Malvern Zetasizer Nano Series V5.10. The concentration of samples used for these measurements was typically corresponding to an absorbance around 0.2 in the plasmon band. Three measurements were usually taken for each sample, each made of 10 to 20 accumulations as optimised by the machine.

### Cell Culture

PC12 cells, were cultured in medium (DMEM supplemented with 10% heat inactivated horse serum, 5% fetal bovine serum, 1% penicillin-streptomycin) @ 37°C and a 5% CO_2 _atmosphere. All the tissue culture plates and chamber slides were treated with 0.001% Poly-L-Lysine (PLL) for 24 hours.

### Cell Staining

Cells were seeded into four-well chambers at a density of 5000 cells/cm^2^. After 24 hours, QDs were added (10% of amount of Medium) to make final concentrations of 10^-9 ^M and the cells were incubated for 17 days. Cells were grown on 4 well Permonax Chamber slides in the presence of QDs and were washed with 1% phosphate-buffered saline (BSA/PBS). Cells were fixed with 4% paraformaldehyde for 15 minutes and then washed 3 times with PBS. Then cells were permeabilized with permeabilizing solution (5 min, 0°C). Actin filaments of cytoplasm were labelled with Phalloidin FITC (Sigma-Aldrich), at 1:50 dilution with PBS for 20 minutes and again washed 3 times with PBS. Nuclei were labelled with Vectashield mounting medium with DAPI to preserve fluorescence and counterstain DNA with DAPI 1 μg/ml.

### Confocal Microscopy

An LSM 510 (Carl Zeiss, Jena, Germany) Confocal Laser Scanning microscope was used to examine QDs inside PC12 cells and their morphology.

Cell imaging was carried out using a LSM 510 Inverted Confocal Microscope which is equipped with the following excitation lasers: (a) Argon Laser excitation wavelengths (λ_Ex_) = 458 nm, 488 nm, 514 nm, (b) HeNe1 - λ_Ex _= 543 nm and (c) Titanium Sapphire Tuneable Two-photon Laser tuneable from 710 nm to 1000 nm with a resulting excitation range of 355 nm to 500 nm.

All Confocal laser scanning was carried out at laser scan speed of 7 with the Photomultiplier Tube settings adjusted to eliminate noise and saturation with the aid of the range indicator setting in the LSM 510 software. For image optimisation, scan averaging was carried out on 8 scans per image.

Sequential acquisition was used to acquire the two-colour images of the QDs in cells. For visualisation of the QDs, the samples were excited with the Argon 514 nm Laser and the microscope configuration was set up to capture the emitted fluorescence at 550 nm or 600 nm as desired. Differential Interference Contrast (DIC) or Nomarski Microscopy was used to visualise the cell morphology, and was carried out by using the HeNe1 488 nm laser with the Transmission Channel Detector selected and the DIC polariser and Nomarski prisms engaged. The two images were then overlaid using the LSM 510 software.

Sequential acquisition was also used to acquire three-colour images. Rhodamine phalloidin was excited using the HeNe1 543 nm laser and the emitted fluorescence was acquired at 575 nm. DAPI stain was excited with laser light at 390 nm (from the Two Photon laser tuned to 780 nm) and emitted fluorescence was acquired at 458 nm. The three separate images were overlaid using the LSM510 software to make up the three-colour images.

### MTT Assay

The yellow tetrazolium MTT (3-(4, 5-dimethylthiazolyl-2)-2, 5 diphenyltetrazolium bromide) is reduced by metabolically active cells, in part by the action of dehydrogenase enzymes, to generate reducing equivalents such as Nicotinamide adenine dinucleotide (NADH) and nicotinamide adenine dinucleotide phosphate (NADPH). The resulting intracellular purple formazan can be solubilized and quantified by spectrophotometry. The MTT Cell Proliferation Assay measures the cell proliferation rate and conversely, when metabolic events lead to apoptosis or necrosis, the reduction in cell viability. PC12 cells of approximately 1000/well were seeded in a flat 96-well micro-plate (Sarstedt) as triplicates. Three different types of controls, namely: positive, negative and background were used throughout the study. Positive control had cells with culture medium treated with NGF but not exposed to QDs. Negative control had QDs without cells. Background control had culture medium without cells. After 24 h, QDs (10% of amount of medium) of size ~ 4.5 nm (red gel, red non-gel) and ~2.5 nm (orange gel and orange non-gel) were added to make final concentrations of QDs to 10^-9 ^M. After 48 hours of seeding, the cells were treated with final concentration of 200 ng/ml of Nerve Growth Factor (NGF) on every second day with 200 μl of fresh medium in each well.

After 10 days of exposure to QDs, old medium was removed from all the wells and 100 μl of fresh medium was added. 10 μl of MTT reagent was then added to each well and incubated for 3 hours. To stop the reaction of the assay, 100 μl of stop solution was added to each well. The 96-well plate was left on a shaker overnight at a speed of 300 rpm and was then analyzed using a Perkin Elmer Victor^3^_TM_V Wallac plate reader at absorbance of 570 nm. This was repeated again for another 96 well plate with incubation period of 16 days after adding QDs [27].

### ApoTox-Glo™Triplex Assay

This combines three assay chemistries to assess viability, cytotoxicity and caspase activation events within a single assay well. In the first part of the assay, it measures two protease activities simultaneously; one being a marker of cell viability and the other being a marker of cytotoxicity. Peptide substrate (glycylphenylalanyl-aminofluorocoumarin; GF-AFC) enters intact cells where it is cleaved by the live-cell protease activity to generate a fluorescent signal proportional to the number of living cells. This live-cell protease becomes inactive upon loss of cell membrane integrity and leakage into the surrounding culture medium. Peptide substrate (bis-alanylalanyl-phenylalanyl-rhodamine 110; bis-AAF-R110) is used to measure dead-cell protease activity, which is released from cells that have lost membrane integrity. Bis-AAF-R110 is not cell-permeable, so no signal from this substrate is generated by intact, viable cells. The live- and dead-cell proteases produce different products, AFC and R110, which have different excitation and emission spectra, allowing them to be detected simultaneously. In the second part of the assay, the Caspase-Glo^® ^3/7 Reagent, added in an "add-mix-measure" format, results in cell lysis, followed by caspase cleavage of the substrate and generation of a "glow-type" luminescent signal produced by luciferase.

PC12 cells of approximately 500/well were seeded in a flat 96-well micro-plate (Sarstedt) as triplicates. Four different types of controls, namely: positive, untreated, negative and background controls were used throughout the study. Positive control had cells with culture medium treated with NGF and exposed to Staurosporine of 500 nM final concentration for 16 hours to induce apoptosis. Control cell cultures contained cells treated with NGF, without QDs. Optional test compound control (negative control) consisted of QDs without cells. No-cell control (background) contained only culture medium without cells. After 24 h, QDs (10% of amount of medium) of size ~ 4.5 nm (red gel, red non-gel) and ~2.5 nm (orange gel and orange non-gel) were added to make final concentrations of QDs to 10^-9 ^M. After 48 hours of seeding, the cells were treated with final concentration of 200 ng/ml of Nerve Growth Factor (NGF) on every second day with 200 μl of fresh medium in each well [27].

After 7 days of exposure to QDs, old medium was removed from all the wells and 100 μl of fresh medium was added. 20 μl of Viability/Cytotoxicity reagent containing both GF-AFC and bis-AAF-R110 substrates was added to each well, and briefly mixed by orbital shaking at 300-500 rpm for 30 seconds and then incubated at 37°C for 30-180 minutes. Fluorescence was measured at 400_Ex_/505_Em _(Viability) and 485_Ex_/520_Em _(Cytotoxicity) by using PerSeptive Biosystems CYTOFLUOR^® ^multi-well plate reader series 4000. After that 100 μl of Caspase-Glo 3/7 reagent was added to each well, and briefly mixed by orbital shaking at 300-500 rpm for 30 seconds and then incubated at room temperature for 30-180 minutes. Luminescence was measured using a Perkin Elmer Victor^3^_TM_V Wallac plate reader by Luminiscence (1.0 s) protocol which is proportional to the amount of caspase activity present. This was repeated again for another 96 well plates with incubation period of 12 and 17 days after adding QDs.

### Statistical Analysis

Results of MTT assay were analysed using one-way analysis of variance (ANOVA). A ρ value of less than 0.05 for the ANOVA was considered significant. Error was expressed as standard deviation.

## Abbreviations

QDs: Quantum Dots; CdTe: Cadmium Telluride; PC12: Pheochromocytoma 12; NGF: nerve growth factor; TGA: Thioglycolic Acid; gel-QDs: gelatinised QDs; DNA: Deoxyribonucleic Acid; DMEM: Dulbecco's Modification of Eagle Medium; EDTA: Ethylenediaminetetraacetic acid; DAPI: 4, 6-diamidino-2-phenylindole; UV: ultraviolet; PL: photoluminescence; PLL: Poly-L-Lysine; BSA/PBS: Bovine serum albumin/phosphate-buffered saline; DIC: Differential Interference Contrast; HBSS: Hank's Balanced Salt Solution.

## Competing interests

The authors declare that they have no competing interests.

## Authors' contributions

BRP performed all the experiments and wrote the manuscript. GM contributed with cellular experiments. VAG, SJB and GLD synthesised QDs and contributed with manuscript preparation. DC contributed with confocal imaging. YR, YG, NN, TJS designed the overall project and helped with data and manuscript revision. All authors read and approved the final manuscript.

## References

[B1] Li KG Chen JT Bai SS Wen X Song SY Yu Q Li J Wang YQ Intracellular oxidative stress and cadmium ions release induce cytotoxicity of unmodified cadmium sulfide quantum dots Toxicol In Vitro 2009 23 6 1007 1013 10.1016/j.tiv.2009.06.020 19540911

[B2] Lovric J Cho SJ Winnik FM Maysinger D Unmodified cadmium telluride quantum dots induce reactive oxygen species formation leading to multiple organelle damage and cell death Chem Biol 2005 12 11 1227 1234 10.1016/j.chembiol.2005.09.008 16298302

[B3] Nel A Xia T Madler L Li N Toxic potential of materials at the nanolevel Science 2006 311 5761 622 627 10.1126/science.1114397 16456071

[B4] Oberdorster G Oberdorster E Oberdorster J Nanotoxicology: an emerging discipline evolving from studies of ultrafine particles Environ Health Perspect 2005 113 7 823 839 10.1289/ehp.7339 16002369 PMC1257642

[B5] Lovrić J Bazzi HS Cuie Y Fortin GRA Winnik FM Maysinger D Differences in subcellular distribution and toxicity of green and red emitting CdTe quantum dots J Mol Med 2005 83 377 385 10.1007/s00109-004-0629-x 15688234

[B6] Byrne SJ Williams Y Davies A Corr SA Rakovich A Gun'ko YK Rakovich YP Donegan JF Volkov Y "Jelly dots": synthesis and cytotoxicity studies of CdTe quantum dot-gelatin nanocomposites Small 2007 3 7 1152 1156 10.1002/smll.200700090 17534993

[B7] Derfus AM Chan WCW Bhatia SN Intracellular delivery of quantum dots for live cell labeling and organelle tracking Advanced Materials 2004 16 12 961 + 10.1002/adma.200306111

[B8] Derfus AM Chan WCW Bhatia SN Probing the cytotoxicity of semiconductor quantum dots Nano Letters 2004 4 1 11 18 10.1021/nl0347334 PMC5588688 28890669

[B9] Lee HM Shin DM Song HM Yuk JM Lee ZW Lee SH Hwang SM Kim JM Lee CS Jo EK Nanoparticles up-regulate tumor necrosis factor-alpha and CXCL8 via reactive oxygen species and mitogen-activated protein kinase activation Toxicol Appl Pharmacol 2009 238 2 160 169 10.1016/j.taap.2009.05.010 19450615

[B10] Conroy J Byrne SJ Gun'ko YK Rakovich YP Donegan JF Davies A Kelleher D Volkov Y CdTe nanoparticles display tropism to core histones and histone-rich cell organelles Small 2008 4 11 2006 2015 10.1002/smll.200800088 18949793

[B11] Duan H Nie S Cell-penetrating quantum dots based on multivalent and endosome-disrupting surface coatings J Am Chem Soc 2007 129 11 3333 3338 10.1021/ja068158s 17319667

[B12] Ruan G Agrawal A Marcus AI Nie S Imaging and tracking of tat peptide-conjugated quantum dots in living cells: new insights into nanoparticle uptake, intracellular transport, and vesicle shedding J Am Chem Soc 2007 129 47 14759 14766 10.1021/ja074936k 17983227

[B13] Cho SJ Maysinger D Jain M Roder B Hackbarth S Winnik FM Long-term exposure to CdTe quantum dots causes functional impairments in live cells Langmuir 2007 23 4 1974 1980 10.1021/la060093j 17279683

[B14] Chan WH Shiao NH Lu PZ CdSe quantum dots induce apoptosis in human neuroblastoma cells via mitochondrial-dependent pathways and inhibition of survival signals Toxicol Lett 2006 167 3 191 200 10.1016/j.toxlet.2006.09.007 17049762

[B15] Foster KA Galeffi F Gerich FJ Turner DA Muller M Optical and pharmacological tools to investigate the role of mitochondria during oxidative stress and neurodegeneration Prog Neurobiol 2006 79 3 136 171 10.1016/j.pneurobio.2006.07.001 16920246 PMC1994087

[B16] Tang M Xing T Zeng J Wang H Li C Yin S Yan D Deng H Liu J Wang M Unmodified CdSe quantum dots induce elevation of cytoplasmic calcium levels and impairment of functional properties of sodium channels in rat primary cultured hippocampal neurons Environ Health Perspect 2008 116 7 915 922 10.1289/ehp.11225 18629314 PMC2453160

[B17] Tang M Wang M Xing T Zeng J Wang H Ruan DY Mechanisms of unmodified CdSe quantum dot-induced elevation of cytoplasmic calcium levels in primary cultures of rat hippocampal neurons Biomaterials 2008 29 33 4383 4391 10.1016/j.biomaterials.2008.08.001 18752844

[B18] Gao X Cui Y Levenson RM Chung LW Nie S In vivo cancer targeting and imaging with semiconductor quantum dots Nat Biotechnol 2004 22 8 969 976 10.1038/nbt994 15258594

[B19] Chan WC Nie S Quantum dot bioconjugates for ultrasensitive nonisotopic detection Science 1998 281 5385 2016 2018 9748158 10.1126/science.281.5385.2016

[B20] Bruchez M Moronne M Gin P Weiss S Alivisatos AP Semiconductor nanocrystals as fluorescent biological labels Science 1998 281 5385 2013 2016 9748157 10.1126/science.281.5385.2013

[B21] Delehanty JB Medintz IL Pons T Brunel FM Dawson PE Mattoussi H Self-assembled quantum dot-peptide bioconjugates for selective intracellular delivery Bioconjugate Chem 2006 17 4 920 927 10.1021/bc060044i PMC2519024 16848398

[B22] Dahan M Levi S Luccardini C Rostaing P Riveau B Triller A Diffusion dynamics of glycine receptors revealed by single-quantum dot tracking Science 2003 302 5644 442 445 10.1126/science.1088525 14564008

[B23] Prasad BR Nikolskaya N Connolly D Smith TJ Byrne SJ Gerard VA Gun'ko YK Rochev Y Long-term exposure of CdTe quantum dots on PC12 cellular activity and the determination of optimum non-toxic concentrations for biological use J Nanobiotechnology 2010 8 7 10.1186/1477-3155-8-7 20338051 PMC2856518

[B24] Greene LA Aletta JM Rukenstein A Green SH PC12 pheochromocytoma cells: culture, nerve growth factor treatment, and experimental exploitation Methods Enzymol 1987 147 207 216 3670084 10.1016/0076-6879(87)47111-5

[B25] Radio NM Freudenrich TM Robinette BL Crofton KM Mundy WR Comparison of PC12 and cerebellar granule cell cultures for evaluating neurite outgrowth using high content analysis Neurotoxicol Teratol 2010 32 1 25 35 10.1016/j.ntt.2009.06.003 19559085

[B26] Mahto SK Yoon TH Rhee SW Cytotoxic effects of surface-modified quantum dots on neuron-like PC12 cells cultured inside microfluidic devices Biochip J 2010 4 1 82 88 10.1007/s13206-010-4113-0

[B27] Pelzl C Arcizet D Piontek G Schlegel J Heinrich D Axonal guidance by surface microstructuring for intracellular transport investigations Chemphyschem 2009 10 16 2884 2890 10.1002/cphc.200900555 19760697

[B28] Yu WW Qu LH Guo WZ Peng XG Experimental determination of the extinction coefficient of CdTe, CdSe, and CdS nanocrystals Chem Mater 2003 15 14 2854 2860 10.1021/cm034081k

[B29] Dawson KA Salvati A Lynch I Nanotoxicology: nanoparticles reconstruct lipids Nat Nanotechnol 2009 4 2 84 85 10.1038/nnano.2008.426 19197306

[B30] Watson P Jones AT Stephens DJ Intracellular trafficking pathways and drug delivery: fluorescence imaging of living and fixed cells Adv Drug Deliv Rev 2005 57 1 43 61 10.1016/j.addr.2004.05.003 15518920

[B31] Byrne SJ le Bon B Corr SA Stefanko M O'Connor C Gun'ko YK Rakovich YP Donegan JF Williams Y Volkov Y Synthesis, characterisation, and biological studies of CdTe quantum dot-naproxen conjugates ChemMedChem 2007 2 2 183 186 10.1002/cmdc.200600232 17177232

[B32] Vu TQ Maddipati R Blute TA Nehilla BJ Nusblat L Desai TA Peptide-conjugated quantum dots activate neuronal receptors and initiate downstream signaling of neurite growth Nano Lett 2005 5 4 603 607 10.1021/nl047977c 15826094

[B33] Gomez N Winter JO Shieh F Saunders AE Korgel BA Schmidt CE Challenges in quantum dot-neuron active interfacing Talanta 2005 67 3 462 471 10.1016/j.talanta.2005.06.041 18970190

[B34] Cooper GM The Cell, A Molecular Approach 2007 Fourth ASM Press Washington, D.C

[B35] Yang Y Xu K Koike T Zheng X Transport of autophagosomes in neurites of PC12 cells during serum deprivation Autophagy 2008 4 2 243 245 18094609 10.4161/auto.5431

[B36] Halliwell B Antioxidant defence mechanisms: from the beginning to the end (of the beginning) Free Radic Res 1999 31 4 261 272 10.1080/10715769900300841 10517532

[B37] Nel A Atmosphere. Air pollution-related illness: effects of particles Science 2005 308 5723 804 806 10.1126/science.1108752 15879201

[B38] Bell AT The impact of nanoscience on heterogeneous catalysis Science 2003 299 5613 1688 1691 10.1126/science.1083671 12637733

[B39] Xiao GG Wang M Li N Loo JA Nel AE Use of proteomics to demonstrate a hierarchical oxidative stress response to diesel exhaust particle chemicals in a macrophage cell line J Biol Chem 2003 278 50 50781 50790 10.1074/jbc.M306423200 14522998

[B40] Oh SH Lim SC A rapid and transient ROS generation by cadmium triggers apoptosis via caspase-dependent pathway in HepG2 cells and this is inhibited through N-acetylcysteine-mediated catalase upregulation Toxicol Appl Pharmacol 2006 212 3 212 223 10.1016/j.taap.2005.07.018 16169029

[B41] Kondoh M Araragi S Sato K Higashimoto M Takiguchi M Sato M Cadmium induces apoptosis partly via caspase-9 activation in HL-60 cells Toxicology 2002 170 (1 2):111-117 10.1016/S0300-483X(01)00498-X 11750088

[B42] Shidoji Y Hayashi K Komura S Ohishi N Yagi K Loss of molecular interaction between cytochrome c and cardiolipin due to lipid peroxidation Biochem Biophys Res Commun 1999 264 2 343 347 10.1006/bbrc.1999.1410 10529366

[B43] Newmeyer DD Ferguson-Miller S Mitochondria: releasing power for life and unleashing the machineries of death Cell 2003 112 4 481 490 10.1016/S0092-8674(03)00116-8 12600312

[B44] McStay GP Clarke SJ Halestrap AP Role of critical thiol groups on the matrix surface of the adenine nucleotide translocase in the mechanism of the mitochondrial permeability transition pore Biochem J 2002 367 Pt 2 541 548 12149099 10.1042/BJ20011672 PMC1222909

[B45] Madesh M Hajnoczky G VDAC-dependent permeabilization of the outer mitochondrial membrane by superoxide induces rapid and massive cytochrome c release J Cell Biol 2001 155 6 1003 1015 10.1083/jcb.200105057 11739410 PMC2150912

[B46] Byrne SJ Corr SA Rakovich TY Gun'ko YK Rakovich YP Donegan JF Mitchell S Volkov Y Optimisation of the synthesis and modification of CdTe quantum dots for enhanced live cell imaging J Mat Chem 2006 16 28 2896 2902 10.1039/b605333e

[B47] Gaponik N Talapin DV Rogach AL Hoppe K Shevchenko EV Kornowski A Eychmüller A Weller H Thiol-capping of CdTe nanocrystals: An alternative to organometallic synthetic routes J Phys Chem B 2002 106 29 7177 7185 10.1021/jp025541k

